# The impact of authoritarian leadership on workplace bullying from the perspective of Chinese Confucian culture: a mediating model with gender as a moderator

**DOI:** 10.3389/fsoc.2025.1586660

**Published:** 2025-07-15

**Authors:** Junjie Yang, Ziyi Luo, Yunxuan Yang, Hanlin Feng

**Affiliations:** Sichuan Agricultural University, Ya’an, Sichuan, China

**Keywords:** authoritarian leadership, hostile work environment, gender, workplace bullying, Confucian culture

## Abstract

Workplace bullying severely impairs employees’ physical and mental health and disrupts the workplace ecosystem. Pinpointing its causes accurately is crucial for effective governance. Drawing on the values of hierarchical order and male dominance over females highlighted in Chinese Confucian culture, and the theoretical framework of the interaction between individuals, environment and behavior (Triadic Reciprocal Determinism), this study takes the hostile work environment as the mediating variable and gender as the moderating variable to explore the influence mechanism of authoritarian leadership on workplace bullying. Data from 1,193 employees were collected through questionnaires, and statistical analyses were conducted using SPSS 29.0 and AMOS software. The results show that authoritarian leadership has a significant positive impact on the occurrence of workplace bullying, and the hostile work environment plays a partial mediating role between them. Meanwhile, compared with men, women are more likely to be targeted by bullying in a hostile environment. This research reveals the profound influence of the concepts of hierarchical order and gender differences in Confucian culture on workplace bullying, and points out the importance of optimizing leadership styles, improving the organizational atmosphere, and paying attention to the vulnerable workplace situation of women in preventing and controlling workplace bullying. The findings provide a theoretical framework for understanding the cultural specificity of workplace behavior and offer gender-differentiated intervention strategies for enterprise management and government policy formulation.

## 1 Introduction

Workplace bullying is a concerning negative phenomenon in the workplace. According to the first global survey report on “Violence and Harassment in the World of Work” released by the International Labor Organization, more than 22% of respondents have experienced workplace violence or harassment.^1^ Coincidentally, China also faces serious workplace injustice. According to statistical data from Zhaopin Limited (ZPIN), 31.2% of white - collar workers in China have experienced workplace bullying.^2^ Multiple studies have shown that workplace bullying not only has a negative impact on employees’ mental health (Jung et al., 2023; Iftikhar et al., 2021), but it also hinders employees’ innovative behaviors and thus reduces organizational performance (Du et al., 2017). Existing research has mainly focused on the negative impacts of workplace bullying on individuals and organizations. There have also been some explorations of antecedent variables. However, interpretations from the perspective of specific regional cultures are lacking.

From the perspective of social psychology, Confucian culture has profoundly shaped the national character of the Chinese people. Inevitably, the workplace context in China is steeped in Confucian culture. Confucian culture emphasizes the hierarchical order of “superiority and inferiority between the upper and lower ranks” (Wang, 2018; Zhang, 2010; Wang and Zhang, 2016), one of which is the idea of “official standard,”^3^ that is, prioritizing the official and regarding the official as valuable and prestigious (Zhu and Bei, 2010). The official standard consciousness in China has a long history and embodies a “submission to the superior” orientation (Ma, 2014). During the more than a 100 years of modern Chinese history, whether it was a series of democratic reforms aimed at saving the Qing Dynasty or the New Democratic Revolution advocating democracy and science, they all attempted to reshape value concepts at their roots. The dissemination of democratic concepts has led to a shift from the traditional hierarchical and unequal social structure to equality for all (Qiu et al., 2018). In terms of cultural and ideological aspects, The People’s Republic of China indeed established a brand-new social system, but history and culture will not cease to exist simply because a new social system emerges. Historical factors such as cultural traditions will be transformed into national psychology – in an non-institutional way - and passed down, which explains why the issue of the bureaucratic system remains a major obstacle in the modernization process of administrative management in contemporary China until today (Luo, 2021).

Specific to the corporate organizational context, leaders, as “superiors,” are the controllers and connectors of various resources. Their behaviors can directly influence employee relationships and power distribution. Leadership style refers to the sum total of relatively fixed and frequently used behavioral methods demonstrated by leaders in organizational management (Miao, 2021). Authoritarian leadership is a directive leadership style that emphasizes the personal authority of leaders, dominance over subordinates, absolute control over subordinates, and requires subordinates to obey unconditionally (Aycan, 2006; Chan et al., 2013). Under the long-term influence of this coercive and highly centralized leadership style, the entire organization may become rather serious and oppressive, causing resistance and rebellion from individuals (Guo et al., 2018), which is not conducive to building a harmonious atmosphere among employees. Triadic Reciprocal Determinism holds that individuals, environments, and behaviors all play a role as determinants. Human behavior is inevitably influenced by social and cultural environments and exhibits unique characteristics different from those in other cultural backgrounds (Hwang, 2015). Driven by the “official-centered” ideology, employees will try to make their words and deeds consistent with those of their superiors to please their superiors, and the upward and downward influence among various levels within the organization leads to a herd effect, undoubtedly accelerating the tension of the organizational atmosphere. Existing studies have shown that authoritarian leadership can lead to negative emotions such as employee silence (Hwang, 2015). As employees gradually lose enthusiasm for their work, this negative emotion will further intensify, manifested as dissatisfaction with the organization and the intentional harm to the legitimate rights and interests of the organization and its stakeholders (Li et al., 2021), ultimately forming a hostile and negative work atmosphere (Clugston et al., 2000), making the relationships among employees relatively cold, which is more likely to trigger conflicts, and then leading to the occurrence of bullying.

The gender concept under Confucian culture emphasizes “men should be in charge of external affairs while women should be in charge of internal affairs” and “there should be a distinction between the inside and outside, with men being superior to women” (Ren, 2020). In the long-term development of feudal hierarchical society, Chinese women have been bound by the four ropes: the power of the state (monarchical power), the power of the gods, the power of the clan, and have become vassals of men (Wang, 2018). Therefore, it is not difficult to imagine that in the organizational environment of China, due to the existing gender hierarchical concepts, women face a despotic working atmosphere and are more inclined to choose to endure oppression and suffer more bullying. Exploring modern workplace issues also requires attention to the changes in modern society. Starting from the New Democratic Revolution and continuing after the founding of the People’s Republic of China, the continuous development of the women’s liberation movement has allowed more women’s strength to rise, gradually forming a Marxist view of women, and the concept of gender equality has been widely spread. When historical origins collide with changes in the times, this study takes the occurrence and prevention of workplace bullying as the entry point to explore whether the concept of gender equality has been manifested in contemporary Chinese society. Therefore, a corresponding empirical analysis is conducted with gender as the moderating variable.

In summary, based on the characteristics of Confucian culture that emphasize hierarchical order and male superiority over females, and in combination with the Triadic Reciprocal Determinism, this study takes the hostile work environment as the mediating variable, constructs a moderated mediating model with authoritarian leadership as the independent variable, workplace bullying as the dependent variable, and gender as the moderating variable, to further explore the occurrence mechanism of workplace bullying in contemporary China.

## 2 Literature review and research hypotheses

### 2.1 Authoritarian leadership and workplace bullying

Authoritarian leadership is one of the unique leadership styles in Chinese enterprise organizations (Chu and Xie, 2012). Zheng conducted in-depth research on the styles of Taiwanese family business leaders and proposed authoritarian leadership, benevolent leadership, and virtue-based leadership (Zheng et al., 2024). Authoritarian leadership is one of the distinctive and important parts of the “trinity model” of paternalistic leadership, referring to the leader emphasizing their absolute authority, being able to control employees and requiring them to obey commands unconditionally (Cheng et al., 2004). In daily management, it specifically includes four behaviors: authoritarian style, belittling subordinates, image enhancement, and instructional behavior (Wu et al., 2002). Reviewing the existing literature, to date, authoritarian leadership is still mostly regarded as a destructive leadership style, which is not conducive to organizational development and employee creativity (Asim et al., 2025; Zhang et al., 2025). It has a significant negative impact on employees’ organizational citizenship behavior (Chan et al., 2013), interpersonal trust (Zheng et al., 2024), and has a significant negative impact on employees’ job happiness (Ma and Zhang, 2018). Although studies have shown that authoritarian leadership can increase employees’ loyalty (Zheng et al., 2024), it still weakens employees’ emotional and cognitive trust in the leader, thereby reducing leadership satisfaction (Zheng et al., 2024). However, existing research mostly focuses on the direct impact of authoritarian leadership on employee behavior, while ignoring its cultural roots. In fact, the cultural tradition of authoritarianism in the Chinese workplace makes authoritarian leadership regarded as a “reasonable” way of exercising power (Guo et al., 2015), and employees have a higher tolerance for leaders’ overstepping authority, indirectly providing a legitimate space for workplace bullying.

Workplace bullying refers to repetitive behaviors with hostility and immorality carried out by one or a few individuals against specific individuals or groups in the workplace. It is characterized by repetition and continuity (Leymann, 1996). Some studies have found that there are many antecedent variables that can lead to the occurrence of workplace bullying, such as hostile workplace interpersonal relationships (Hauge et al., 2007), stress (Neuman et al., 2003), and stimuli generated by team and organizational characteristics (De Cuyper et al., 2009). The lack of appropriate leadership behavior is an important influencing factor that induces the occurrence of workplace bullying (Fu et al., 2013). In addition, some scholars have pointed out that the root cause of workplace bullying is a power issue. Bullying usually occurs when there is a power imbalance between the perpetrator and the target (Matthiesen and Einarsen, 2001; Mikkelsen and Einarsen, 2001). It is worth noting that the required power disparity can also occur among colleagues at the same level. In some cases, even subordinates, especially when acting in a team, may have enough power to bully their supervisors (Salin, 2003). At the same time, the greater the status power that leaders possess in an organization, the more authority they demonstrate, and the more obedient employees will be (Chen et al., 2014). The pressure to obey and the fear and vigilance that arise under such pressure (Aryee et al., 2007) will increase employees’ psychological stress, leading to a sense of frustration. Frustrated employees, on the contrary, will blame each other and become a source of stress for one another, ultimately leading to the occurrence of workplace bullying (Leymann, 1996).

Based on the above analysis, this study proposes the following hypothesis:

*H1*: Authoritarian leadership has a significantly positive impact on workplace bullying.

### 2.2 The mediating role of the hostile work environment

#### 2.2.1 Authoritarian leadership and hostile work environment

A hostile atmosphere is a specific emotional climate that naturally forms when employees perceive jealousy, mistrust, and aggressive attitudes (Mawritz et al., 2012). In situations where highly authoritarian and autocratic leadership styles prevail, a hostile atmosphere is particularly common (Aryee et al., 2007; Karakitapoğlu-Aygün et al., 2023). Authoritarian leaders tend to adopt a more distant approach in interactions (Chan et al., 2013), thus creating a hostile work environment within the team, that is, an emotional organizational atmosphere filled with mistrust, suspicion, and confrontation (Mawritz et al., 2014). In such an environment, team members are more likely to exhibit negative emotions such as anger, fear, hostility, and mistrust. These emotions weaken the positive interactions among team members and have an adverse impact on the social functions of the team (Zahlquist et al., 2023; Mackey et al., 2021; Peng et al., 2014). At the individual level, employees who are exposed to a hostile work environment for a long time will experience feelings of frustration or apathy, which will further lead to psychological problems such as emotional exhaustion, anxiety, and depression (Sliter et al., 2012; Korunka et al., 2008). At the group level, an organizational hostile atmosphere will lead to a decrease in employees’ social activities, a decline in group dependence, and a weakening of the organizational citizenship behavior and willingness to cooperate among group members (Ng et al., 2023; Liu and Yu, 2017), that is, the team cohesion is low, and the relationships among employees are cold and full of mistrust.

#### 2.2.2 Hostile work environment and workplace bullying

When studying the antecedents of workplace bullying, most research has focused on more direct work - related factors that individuals directly experience, such as employees experiencing role conflicts or high and heavy workloads, as well as their perception of the leadership style of their immediate superiors (Agotnes et al., 2018). However, situational risk factors may also exist at different levels of the organization and affect the occurrence of workplace bullying behavior. Previous studies have shown that the work environment plays an important role in the risk of workplace bullying (Salin and Hoel, 2020). According to the work environment hypothesis (Leymann, 1996; Einarsen et al., 1994), bullying is mainly caused by organizational deficiencies in work design, leadership practices, a hostile social atmosphere in workgroups, and a culture that allows or even rewards such bullying behavior (Einarsen et al., 2020). Specifically, the tripartite model proposed by Baillien et al. (2009) elaborates on the occurrence mechanism of workplace bullying from the work environment level: Deficiencies in the work environment may increase the risk of bullying by triggering conflicts that may escalate into bullying, causing frustration that leads to aggressive behavior, or directly contributing to or stimulating bullying behavior. Such a work environment filled with emotions of annoyance, frustration, and aggression (Bandura, 1977; Stapinski and Gamian-Wilk, 2024) can form a behavioral demonstration effect through the attention process theory (Salancik and Pfeffer, 1978) - when negative behaviors are repeatedly observed and not punished, employees will internalize them as acceptable behavior patterns (Maneethai, 2019). Empirical studies have also confirmed that such a relatively hostile climate not only provides psychological incentives for negative behaviors (Jones and James, 1979), but also directly constitutes a breeding ground for bullying behavior (Rosander and Salin, 2023; Salin and Hoel, 2020).

Based on the above analysis, this study proposes the following hypothesis:

*H2*: The hostile work environment plays a mediating role between authoritarian leadership and workplace bullying.

### 2.3 The moderating role of gender

Confucian culture, as the core pillar of China’s social ethical system, emphasizes the social division of labor and role expectations between men and women (Zhang, 2010). In the workplace, this cultural tradition is embodied in the differentiated construction of gender-specific behavioral patterns: Men are endowed with the social role of “vigorous and proactive,” and are required to demonstrate both authoritative and enterprising traits within the rigid framework of “not being swayed by wealth, not being moved by poverty, and not being subdued by power” (Wan and Lan, 2012); Women are disciplined as the carriers of “gentle and obedient,” maintaining interpersonal harmony through the flexible traits of “mercy, leniency, and fairness” (Holt-Lunstad, 2018; Yu, 2016; Wang, 2011). Although the awareness of gender equality in modern society has gradually increased, the continuity of cultural genes may still subtly influence the workplace interaction logic through Confucian gender concepts. In workplace bullying research, some researchers report bullying as a neutral phenomenon (Rosander et al., 2020; Tsuno et al., 2015), but there is also evidence that the proportion of female victims is higher (Zapf et al., 2020; Salin, 2021). The theory of social roles (Eagly and Mitchell, 2004; Eagly and Wood, 2013) indicates that social culture shapes individual behavioral expectations through gender role stereotypes. The particularity of the Chinese cultural context lies in that under the suppression of thousands of years of feudal ethics, Chinese women have accumulated historical humiliation and suffering, internalized traditional moral concepts, and formed a unique Eastern female temperament: restraint, perseverance, subtlety, and gravity (Wang, 2018), which together with Confucian “harmony is precious”^4^ training has shaped a unique coping mechanism - “tolerance.” This unique female tolerance trait and the passive response to interpersonal conflicts in the organization may make them more likely to become the target group of bullying under the obedience pressure created by hostile working atmosphere.

Based on the above cultural mechanisms and theoretical logic, this study believes that gender is not a simple binary variable, but will affect the causal relationship strength of “hostile working atmosphere → workplace bullying” by regulating individuals’ perception patterns and coping strategies toward hostile environments. Therefore, the following research hypothesis is proposed:

*H3*: Gender plays a moderating role in the relationship between hostile work environment and workplace bullying. Compared to male employees, the positive impact of hostile work environment on workplace bullying is more significant for female employees.

**Figure 1:**
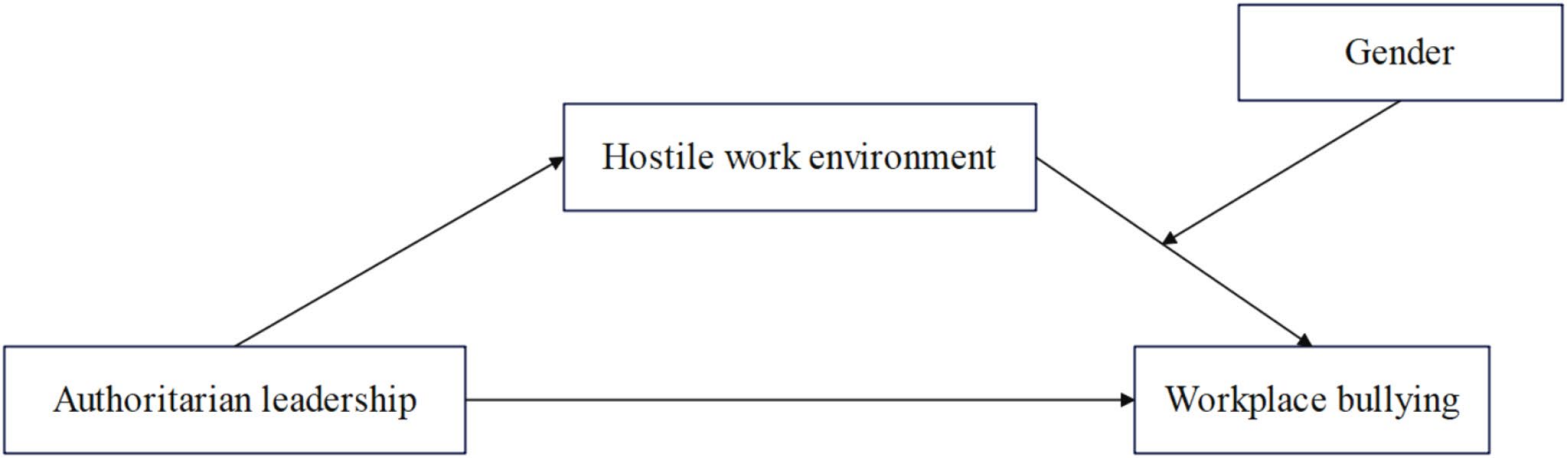
The theoretical model diagram constructed in this study.

Based on the above analysis, the following research model diagram can be constructed (Figure 1).

## 3 Research design

### 3.1 Research methods

This study uses the questionnaire survey method to collect data and explores the influence mechanism of authoritarian leadership on workplace bullying through quantitative analysis. SPSS 29.0 is used for descriptive statistics and correlation analysis, and the PROCESS 4.1 plugin is employed to test the moderating effect of gender. The significance of the moderated mediation model is verified by the Bootstrap method (with 10,000 repeated samplings and a 95% confidence interval). In addition, AMOS software is used to establish multiple groups of structural equation models (SEM) to test the mediating effect of the hostile work environment between the independent variable and the dependent variable. The significance of the partial mediating effect is verified through path analysis combined with model fit indices.

### 3.2 Data collection

The data for this study were collected through a questionnaire survey. Before the formal survey, the research team conducted a pre-survey among 50 employees from a private enterprise in Sichuan Province, China. Based on the results of the pre-survey, the structure and wording of the questionnaire were revised, and its reliability and validity were preliminarily tested. Then, the research team used the revised questionnaire to conduct a formal survey, and the survey scope covered various companies, enterprises and other organizations in Sichuan Province, China. In the process of sample selection, this study ensured that all in-service employees had an equal chance of being selected, that is, the entire group of in-service employees was covered without specific restrictive conditions. In order to overcome the sample bias caused by online dissemination and convenience sampling, first of all, in the questionnaire design, the research team used mature scales with high reliability and validity (Table 1), and made efforts to ensure that during the translation process, the item statements conformed to the Chinese expression habits. Secondly, the research team strived to expand the sample size, reduce errors, and improve representativeness. A total of 1,300 questionnaires were distributed through on-site visits, emails, and social media platforms. A total of 1,252 questionnaires were actually recovered. After excluding questionnaires with incomplete filling and logical errors, 1,193 valid questionnaires were retained, with an effective recovery rate of 91.78%. The sample characteristics are shown in Table 2 (in order to better observe the gender differences in the sample, the data were subdivided).

**Table 1:** Sources, content descriptions, and reliability and validity tests of the measurement scales.

Variable	Source	Representative items	Cronbach’s α coefficient	KMO measure
AL	Zheng et al. (2024)	He requires me to completely obey his leadership (a total of 5 questions.)	0.695	0.780
HWE	Rosander and Blomberg (2018)	The characteristics of my workplace are suspicion, conflict, misunderstanding, and rudeness (a total of 5 questions.)	0.693	0.734
WB	Notelaers and Einarsen (2019)	Someone has concealed information that would affect my work performance (a total of 9 questions.)	0.878	0.932

AL, Authoritarian Leadership; HWE, Hostile Work Environment; WB, Workplace Bullying.

**Table 2:** Distribution of the sample (make a secondary classification based on gender, where male = 0 and female = 1).

Variable	Gender	Frequency	Percentage (%)
Age			
18–25	0	149	26.70
	1	215	33.90
26–35	0	165	29.50
	1	146	23.00
36–45	0	137	24.50
	1	141	22.20
Over45	0	108	19.30
	1	132	20.80
Education			
Primary school and below	0	7	1.30
	1	18	2.80
Junior high school	0	37	6.60
	1	72	11.40
Senior high school	0	88	15.70
	1	101	15.90
Bachelor’s degree	0	343	61.40
	1	357	56.30
Master’s degree	0	70	12.50
	1	77	12.10
Doctoral degree	0	14	2.50
	1	9	1.40
Industry			
Manufacturing	0	59	10.60
	1	33	5.20
Finance and insurance	0	49	8.80
	1	54	8.50
Service industry	0	63	11.30
	1	64	10.10
Wholesale and retail trade	0	37	6.60
	1	21	3.30
Computers and internet	0	44	7.90
	1	26	4.10
Healthcare	0	35	6.30
	1	42	6.60
Agriculture	0	24	4.30
	1	30	4.70
Food industry	0	21	3.80
	1	19	3.00
Engineering and design	0	47	8.40
	1	38	6.00
Legal services	0	15	2.70
	1	8	1.30
Education	0	38	6.80
	1	63	9.90
Arts	0	6	1.10
	1	10	1.60
Social work	0	59	10.60
	1	33	5.20
Others	0	49	8.80
	1	54	8.50
Amount of Staff			
0–100	0	258	46.20
	1	363	57.30
101–500	0	167	29.90
	1	125	19.70
501–1,000	0	72	12.90
	1	52	8.20
Over1000	0	62	11.10
	1	94	14.80
Type of work unit			
State-owned business	0	103	18.40
	1	126	19.90
Private enterprise	0	222	39.70
	1	259	40.90
Joint venture enterprise	0	36	6.40
	1	16	2.50
Foreign-owned enterprise	0	41	7.30
	1	21	3.30
Public institution	0	97	17.40
	1	126	19.90
Governmental agencies	0	60	10.70
	1	86	13.60
Work experience			
Less than 1 year	0	91	16.30
	1	165	26.00
1–3 years	0	125	22.40
	1	153	24.10
3–5 years	0	124	22.20
	1	83	13.10
5–10 years	0	114	20.40
	1	107	16.90
Over 10 years	0	105	18.80
	1	126	19.90
Position level			
Senior management	0	44	7.90
	1	59	9.30
Middle management	0	143	25.60
	1	104	16.40
Front-line management	0	131	23.40
	1	113	17.80
Regular employee	0	241	43.10
	1	358	56.50

### 3.3 Variable measurement

This study mainly involves three continuous variables: authoritarian leadership (AL), hostile work environment (HWE), and workplace bullying (WB). The entire questionnaire adopts a five-point Likert scale, with scores ranging from low to high, assigned as “1–5.” In order to make the measurement tool adapt to the Chinese cultural context and the characteristics of this study, on the basis of referring to existing mature scales, this study modified the items that are prone to ambiguity due to different communication methods to make them suitable for the Chinese context. Finally, the back-translation method was used to check the semantics. For example, “When you approach others, you face hostile reactions” was changed to “When I approach others, they show hostility toward me.”

Authoritarian leadership mainly refers to the paternalistic leadership scale used in Zheng et al. (2024) research. For example, “He requires me to completely obey his leadership.” and “When I oppose him in public, I will be met with sarcastic remarks.” etc.; The measurement of the hostile work environment uses a 5-item scale from the Psychosocial Work Environment Questionnaire (Rosander and Blomberg, 2018), such as “The characteristics of my workplace are suspicion, conflict, misunderstanding, and rudeness.” and “There are ongoing conflicts in my workplace.” etc.; The measurement of workplace bullying adopts the S-NAQ questionnaire modified from Notelaers and Einarsen (2019), such as “Someone has concealed information that would affect my work performance.” and “Spreading rumors about me.” etc.

The Cronbach’s Alpha coefficient was used to conduct a reliability test on the above variables. The Cronbach’s coefficients of authoritarian leadership, hostile work environment, power distance, and workplace bullying were 0.695, 0.693 and 0.878 respectively, indicating good internal consistency. The structural validity among the variables was verified through the Kaiser-Meyer-Olkin (KMO) test and Bartlett’s test of sphericity. The results showed that the KMO values of the above variables were 0.780, 0.734 and 0.932, respectively. All the above KMO values were above 0.6, indicating that it is quite suitable for information extraction and the validity is good.

## 4 Empirical analysis

### 4.1 Common method bias test

In order to avoid common method bias, this study adopted procedural control methods such as anonymous questionnaire measurement and setting reverse-scoring items. The Harman single-factor test was used to test the common method bias of the collected data. It was found that the results of the exploratory factor analysis without rotation extracted a total of 4 factors with eigenvalues >1. The variance explanation rate of the largest factor was 32.594%, which did not reach the critical value of 40%. This indicates that there is no serious common method bias in this study.

### 4.2 Mean statistics and correlation analysis of AL, HWE, and WB

The statistical results show (Table 3) that the overall mean value of authoritarian leadership (AL) is 2.877, which is slightly above the medium level. This indicates that the leadership image in the Chinese workplace context is relatively authoritative and emphasizes obedience, reflecting the common cultural pattern of the Chinese workplace. The overall mean value of the hostile work environment (HWE) is 2.101, which is at a medium-low level, indicating that most of the measured samples have a relatively comfortable, harmonious and trusting work environment, but there are also some disharmonious factors. The overall mean value of workplace bullying (WB) is 1.522, which is basically the same as the international general level of 1.5 (Notelaers and Einarsen, 2019), indicating that for the research samples, workplace bullying is prevalent, but it has not reached a serious level.

**Table 3:** Mean statistics and correlation coefficient matrix of AL, HWE, and WB.

Variable	M ± SD	AL	HWE	WB
AL	2.877 ± 0.852	1	0.439***	0.467***
HWE	1.522 ± 0.574	0.444***	1	0.596***
WB	2.101 ± 0.770	0.473***	0.610***	1

AL, Authoritarian Leadership; HWE, Hostile Work Environment; WB, Workplace Bullying. ***P < 0.001 indicates a significant correlation.

As shown in the table, the lower triangular part of the correlation coefficient matrix is the ordinary correlation coefficient (r), and the upper triangular part is the partial correlation coefficient (pr) with control variables such as age, gender, education level, years of service, and the Type of work unit added. The ordinary correlation coefficients show that there are significant positive correlations between authoritarian leadership (AL) and the hostile work environment (HWE) (r = 0.444, *p* < 0.01) and workplace bullying (WB) (r = 0.473, *p* < 0.01), and the correlations are of moderate strength. There is also a significant positive correlation between HWE and WB (r = 0.610, *p* < 0.01), and the correlation is of relatively high strength. In addition, after adding a series of control variables, the partial correlation coefficients do not change significantly and remain significant, further indicating the independent relationships between the variables, which are not spurious correlations. Therefore, regression analysis can be further carried out.

### 4.3 Hypothesis testing

#### 4.3.1 Mediation effect testing

**Figure 2:**
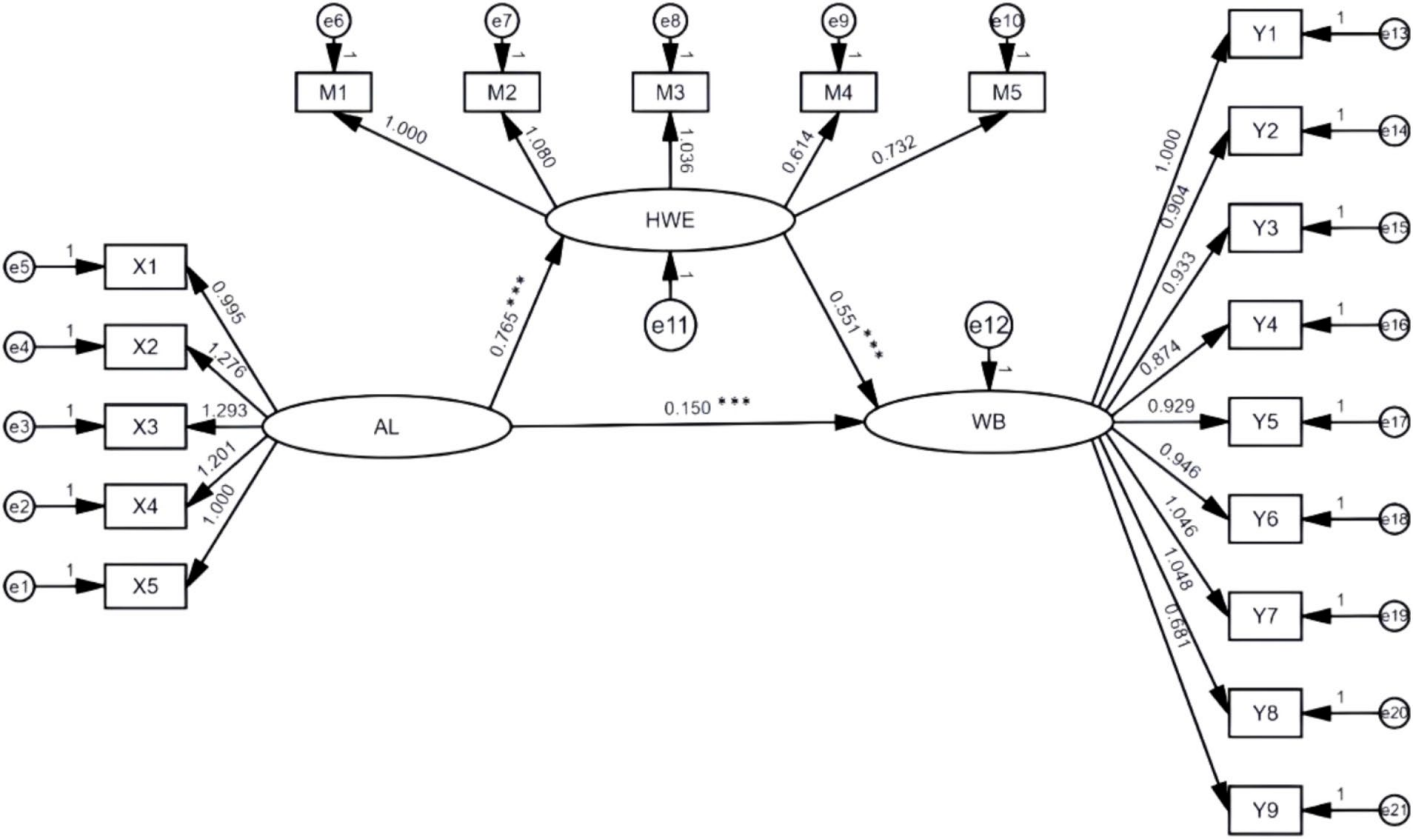
Path coefficients of the structural equation model. ***p < 0.001; AL, Authoritarian Leadership; HWE, Hostile Work Environment; WB, Workplace Bullying.

Based on the mediation effect test procedure for structural equations proposed by Wen and Ye (2014), using the bias-corrected non-parametric percentile Bootstrap method, 5,000 samples were randomly selected repeatedly to estimate the confidence intervals of each coefficient. The first step was to test the direct effect of authoritarian leadership on workplace bullying. The results showed that the model fit was good, with RMSEA = 0.033, TLI = 0.977, and CFI = 0.981 (see Table 4). The positive predictive effect of authoritarian leadership on workplace bullying was significant (*β* = 0.607, *p* < 0.001), and Hypothesis 1 is verified. According to Cohen (1988) standard, this effect size (*β* > 0.5) belongs to a large effect, indicating that for every one standard deviation increase in authoritarian leadership, the workplace bullying score is expected to increase by 31%^5^ of its mean value. Step 2: add the hostile work environment as an mediating variable to the original model. The results show that RMSEA = 0.036, TLI = 0.960, CFI = 0.965 (as shown in Table 4), all the fitting index values meet the judgment criteria, and the structural equation model is compatible with the data. On this basis, the path coefficients of each path in the mediating model are estimated using the maximum likelihood method (ML), as shown in Figure 2. Authoritarian leadership has a positive predictive effect on workplace bullying (*β* = 0.150, *p* < 0.001), authoritarian leadership has a positive predictive effect on hostile work atmosphere (*β* = 0.765, *p* < 0.001), and hostile work atmosphere has a positive predictive effect on workplace bullying (*β* = 0.551, *p* < 0.001).

**Table 4:** Model fitting comparison table.

Model fit	Recommended value	Final value 1	Final value 2	References
RMSEA	<0.07	0.033	0.036	Steiger (2007)
TLI	>0.09	0.977	0.960	Bollen (1989)
CFI	>0.09	0.981	0.965	Bagozzi and Yi (1988)

Final value 1 represents the fitting index values of the direct effect model; Final value 2 represents the fitting index values of the mediation effect model.

As shown in Table 5, the direct effect of authoritarian leadership on workplace bullying is significant, with a 95% confidence interval of [0.058, 0.244], excluding 0. The indirect effect of authoritarian leadership on workplace bullying is 0.421, with a 95% confidence interval of [0.324, 0.553], excluding 0. The research results indicate that the hostile work atmosphere has a partial mediating effect between authoritarian leadership and workplace bullying, and Hypothesis 2 is verified.

**Table 5:** Decomposition table of the mediation effect of the hostile work environment.

Effect indicators	Effect	BootSE	BootLLCI-95%	BootULCI-95%
Total effect	0.571	0.059	0.464	0.699
Direct effect	0.150	0.047	0.058	0.244
Indirect effect	0.421	0.057	0.324	0.553

In conclusion, the following path coefficient diagram of the structural equation model can be drawn.

#### 4.3.2 Moderation effect testing

The moderation effect of gender was tested using Model 14 in PROCESS 4.2. The test results are shown in the table. After controlling variables such as education level and age, the interaction term between the hostile work environment (HWE) and gender had a significant predictive effect on workplace bullying (WB) (*β* = 0.088, *p* < 0.01), and the corresponding 95% confidence interval (CI) was [0.022, 0.154]. This indicates that gender plays a moderating role between the hostile work environment and workplace bullying, and the moderated mediation model is significant, thus verifying Hypothesis 3 (Table 6).

**Table 6:** Test of the moderated mediation model.

Regression equation		fitting index		Regression coefficient	
Outcome variable	predictor variable	R^2^	F	*β*	*t*
HEW	AL	0.198	293.260***	0.401	17.125***
WB	AL	0.428	222.083***	0.172	10.399***
	HWE			0.227	3.960***
	Gender			−0.141	−1.899
	HWE × Gender			0.088	2.630**

AL, Authoritarian Leadership; PD, Power Distance; HWE, Hostile Work Environment; WB, Workplace Bullying. **p < 0.01; ***p < 0.001.

**Figure 3:**
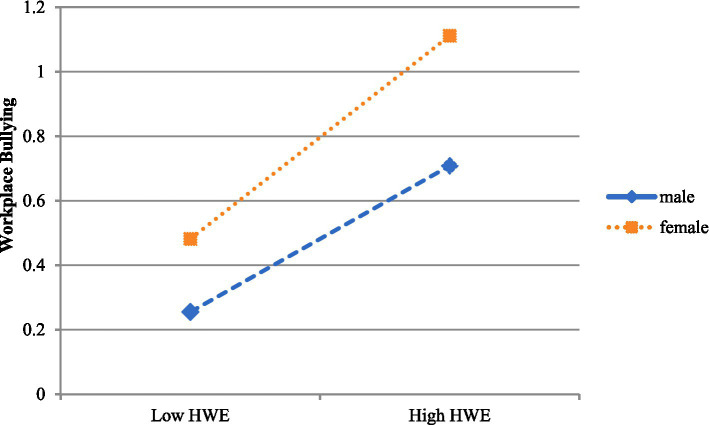
The moderating effect of gender on the influence of the HWE on WB. HWC, Hostile Work Environment; Low HWC is equal to −1 SD and high HWC is equal to +1 SD.

In order to further clarify the specific influence of the moderating effect of gender, a simple slope test was carried out (Figure 3). The results show that in terms of the impact of the hostile work environment on workplace bullying, whether for male employees (*β* = 0.315, t = 11.410, *p* < 0.001) or female employees (*β* = 0.403, t = 17.990, *p* < 0.001), as the perception of the hostile work environment increases, the frequency of workplace bullying shows an increasing trend. Moreover, compared with male employees, the hostile work environment has a greater predictive effect on workplace bullying experienced by female employees. This indicates that in a hostile, conflicting, and distrustful work environment, women are more likely to suffer from workplace bullying.

## 5 Discussion

This study has three main findings: (1) authoritarian leadership has a significant positive impact on workplace bullying (H1); (2) the hostile work environment plays a mediating role between authoritarian leadership and workplace bullying (H2); (3) gender plays a moderating role in the impact of the hostile work environment on workplace bullying, and women are more affected than men (H3).

Authoritarian leadership significantly positively predicts workplace bullying. This is consistent with previous research results (Vartia, 1996), which indicates that when leaders demonstrate greater authority, interpersonal interactions among employees tend to become more hostile, thereby increasing the likelihood of workplace bullying incidents. Authoritarian leaders tend to act in their own way, emphasizing “personal domination” over subordinates and concentrating authority on themselves. This strong desire for control over subordinates and the need for power leads them to be more likely to engage in workplace bullying behavior (Ashforth, 1997). Moreover, autocratic leaders often lack effective emotional management skills, which further increases their risk of exhibiting bullying behavior in the workplace (Aryee et al., 2007; Ashforth, 1997). It should be noted that this study focuses on the “autocratic” dimension of authoritarian leadership (Zhou et al., 2010), which is mainly characterized by controlling people and requiring absolute obedience from employees. As for the “strictness” dimension, it has not been involved in this study. It focuses on work tasks, and relevant studies have shown that this dimension will have a positive impact on employees’ behavior (Maqsoom et al., 2022; Zhang and Sun, 2020). Longitudinal comparisons reveal that the effect size of authoritarian leadership on workplace bullying in this study (*β* = 0.607) is significantly higher than that of international similar studies (such as the *β* value reported by Ertureten et al., 2013, which is 0.35), possibly due to the strengthening of the legitimacy recognition of power imbalance by Chinese Confucian culture. In China, in the workplace dominated by the concept of “official standard,” it can be summarized by two words: “obeying the top” and “flattery.” Authoritarian leaders undoubtedly become the “catalyst” of the official supremacy ideology. The leader requires absolute obedience from subordinates, and subordinates become more profit-driven to please; the leader is harsh and highly controlling toward subordinates, resulting in upward and downward imitation among them, a serious and rigid workplace atmosphere, full of conflicts, and ultimately leading to frequent bullying incidents.

The hostile work environment has a partial mediating effect on the influence of authoritarian leadership on workplace bullying. The high pressure exerted by authoritarian leadership not only increases the frequency of workplace bullying, but this study also finds that the behavior of authoritarian leadership can also lead to workplace bullying by giving rise to a hostile work environment. This finding once again validates the work environment hypothesis, that is, bullying mainly stems from environmental and cultural deficiencies (Einarsen et al., 2003). Authoritarian leaders are accustomed to strengthening their control over subordinates by virtue of their authority. They are highly punitive, approve of the strong and reject the weak, thus creating a tense and hostile atmosphere and an environment where the strong bully the weak. Based on Social Learning Theory (Bandura, 1977), the environment can influence employees’ behavior patterns and cognitive structures. Moreover, Keashly et al. (2020) also found through a review of relevant studies that the organizational climate may affect the risk of harassment and aggressive behavior. In the organizational context of a hostile work environment, the distrust, suspicion, and conflicts in the workplace will gradually escalate and then evolve into workplace bullying. This finding is consistent with previous research results (Blomberg et al., 2024; Rosander and Salin, 2023).

Gender plays a moderating role in the positive influence of the hostile work environment on workplace bullying, and women are more affected than men. This indicates that in a work context full of confrontation, suspicion, and mistrust, people often direct the conflict more toward women, making them the target for venting emotions. Existing research has shown that there are differences in stress regulation between genders (Loh and Snyman, 2020; Rosander and Salin, 2023), and gender may moderate the relationship between stressors and outcomes (Loh and Snyman, 2020). This provides a theoretical explanation for why gender plays a moderating role in the influence of the hostile work environment (a stressor) on workplace bullying (a behavioral outcome). From the perspectives of gender and culture, feminist researchers have proposed that women are often assigned to specific gender roles and stereotypically categorized as the vulnerable group (McLaughlin et al., 2017), and deviating from cultural norms may invite bullying from men (Mittleman, 2023). In China, although the family role and division of labor model of “men dominate outside, women dominate inside” advocated by Confucianism was somewhat compatible with the social productivity level of China’s traditional agricultural society at that time (Yu, 2016; Zhang et al. 2023), it has to a certain extent imprisoned people’s thoughts and behaviors. The influence of thousands of years of traditional gender culture has deeply engraved the mark of gender preference in people’s subconsciousness, and it has developed into a psychological habit and social custom with strong vitality and cultural characteristics. Although long-term publicity and education on gender equality have enabled most people to establish the gender concept of equality between men and women in their subjective consciousness, there are still some unequal behavior patterns in real life, showing a phenomenon of inconsistency between ideology and behavior (Lv, 2010). This also explains why women still encounter more differential treatment and become the bullied party in the workplace in the 21st century. It is worth noting that the greater vulnerability of women to workplace bullying is not a problem unique to China. When Connie Zheng et al. (2024) studied the issue of workplace bullying of female employees in Pakistan, they found that developing countries such as Uganda, India, and Bangladesh also face the same problem.

## 6 Implications

### 6.1 Theoretical significance

At the theoretical level, this study deepens the understanding of the generation mechanism of workplace bullying, especially focusing on its uniqueness within the context of Chinese Confucian culture. Previous studies mostly took Western culture as the paradigm and failed to fully consider the shaping effect of social value orientations such as hierarchical concepts, authority worship, and gender preference contained in Confucian culture on workplace behavior patterns. By constructing an integrated theoretical model that includes moderating variables and mediating variables, this study systematically expounds on the triggering and transmission mechanism of authoritarian leadership style on workplace bullying phenomena within the field of Confucian culture, achieving an innovative expansion of the existing theoretical system. The study finds that there is a complex interactive relationship between authoritarian leadership and workplace bullying. Under the cognitive framework of Confucian culture that emphasizes “order of superiority and inferiority” and “obedience to authority,” this relationship is strengthened through the psychological contract mechanism of “authority-compliance.” This finding not only reveals the deep logic of the interaction between leadership behavior and workplace behavior, but also constructs a theoretical analysis framework that includes the analysis of cultural variables.

In terms of the gender dimension, this study confirms the moderating effect of gender differences on the susceptibility to workplace bullying, opening up a new path for research in this field. The study shows that female employees are significantly more at risk of experiencing workplace bullying in the context of authoritarian leadership than male employees. This phenomenon can be explained by the gender role socialization theory (Eagly, 1987): the gender stereotypes and the perception of power distance in traditional culture make women more likely to have an obedient response when facing authority, thus increasing the probability of workplace bullying. This finding provides confirmatory conclusions for subsequent research on interventions against workplace bullying based on gender differences.

### 6.2 Practical significance

#### 6.2.1 For enterprises

This study reveals that authoritarian leadership significantly exacerbates workplace bullying through the mediating path of a hostile work environment, indicating that enterprises need to focus on optimizing organizational culture to mitigate the negative effects of authoritarian leadership. Firstly, management mechanisms should be reconstructed to reduce conflicts arising from authoritarian behavior. For example, implementing a distributed decision-making model (such as a dual-channel promotion system combining technical and managerial tracks). This approach can draw on the balancing strategies of “ethical leadership” in the theory of paternalistic leadership (Zheng et al., 2024), reducing one - way control of leaders over employees through decentralization. Secondly, targeted emotional management training programs should be designed, especially focusing on the “autocratic style” dimension of authoritarian leadership (Wu et al., 2002). Empathy and conflict - resolution skills of managers can be enhanced through situational simulations. The mean value of the hostile work environment in this study (M = 2.101) indicates that latent conflicts already exist in some enterprises, and such training can effectively prevent the deterioration of the hostile atmosphere.

When drawing on international experience, a cautious and critical attitude is required. For instance, although the third - party supervision mechanism of WorkSafeBC in Canada can independently intervene in bullying incidents, directly transplanting it into the Chinese context may face challenges in cultural adaptability. Chinese collectivistic culture relies more on internal negotiation (Hofstede, 2001). It is recommended to integrate third - party supervision into the existing functions of trade unions, and achieve localization improvement through a “joint committee of trade unions and management.”

In addition, a gender - sensitive prevention and control system needs to be established. This study finds that the hostile work environment has a stronger predictive effect on bullying of female employees (*β* = 0.403*** vs. *β* = 0.315*** for male employees). Therefore, enterprises should conduct gender - inclusive leadership training, incorporate “unconscious bias detection” into the manager evaluation system, and correct gender stereotypes in authoritarian behavior through role - playing (such as avoiding defaulting female employees as submissive roles). Meanwhile, anti - bullying departments independent of the hierarchical structure can be set up, staffed with specialists with a background in gender studies to distinguish the complex forms of “power - based bullying” and “gender - based bullying.” Regular anonymous monitoring of gender differences in the hostile atmosphere should be carried out, and mandatory intervention procedures should be initiated for unbalanced departments.

#### 6.2.2 For government

The government needs to construct a multi - level intervention framework based on empirical evidence. This study found that authoritarian leadership has both direct and indirect effects on workplace bullying. Therefore, it is necessary to clearly define the operational definition of “workplace bullying” at the legal level. Referring to the scale items of Notelaers and Einarsen (2019), criteria for determining “sustained hostile behavior” (such as criticism frequency ≥ 3 times/week and resulting in a psychological diagnosis certificate) can be incorporated into the revision of the Labor Law to distinguish between normal management behavior and bullying. However, considering the prevalence of authoritarian leadership in China’s workplace culture (the mean value of AL is M = 2.877), directly implementing individualism - oriented laws may encounter resistance in enforcement. It is recommended to adopt a “pilot - diffusion” strategy: first implement the anti - bullying standards of the International Labor Organization (ILO) in multinational companies, and then gradually promote them to local enterprises through the restructuring of state - owned enterprises to achieve cultural buffering.

Policy design should incorporate the perspective of gender mainstreaming. For example, a “work environment safety” clause could be added to the Law on the Protection of Women’s Rights and Interests, requiring enterprises with more than 300 employees to publish annual reports on gender - based bullying and include this in the ESG rating system. This measure can echo the gender moderating effect of the hostile atmosphere found in this study and also encourage enterprises to establish cross - sectional complaint channels. In addition, universities and NGOs can be jointly mobilized to develop a “workplace bullying risk assessment toolkit,” integrating scales for authoritarian leadership, hostile work environment, and workplace bullying, to provide free diagnostic services for enterprises. At the international governance level, the ILO Convention on Violence and Harassment in the World of Work (C190) can be referred to, but its individualistic clauses need to be adjusted. For example, “anonymous reporting” can be changed to “union - representative reporting” to conform to China’s tradition of collective negotiation. Labor inspection departments also need to receive training in cultural psychology to identify implicit authoritarian behaviors rationalized by Confucian discourse on “order of superiority and inferiority” (such as “a strict teacher produces outstanding students”), thereby enhancing the practicality of law enforcement.

## 7 Conclusion

This study, from a theoretical perspective, analyzed workplace phenomena based on the specific cultural background of a certain region, revealing the intricate interplay between gender and culture, which is unique. In practice, it proposed distinctive measures specific to the region and also extracted innovative measures applicable globally, which are conducive to promoting workplace ecological governance and high-quality employment, and further contribute to the protection of women’s rights. Although the research tried to be systematic and complete, and provide sufficient arguments, it still needs to acknowledge some issues: Firstly, it may have missed some other explanatory variables that affect workplace bullying behaviors, such as job insecurity and personality traits; Secondly, due to the complexity of China’s 5000-year cultural and institutional environment, discussions related to historical and cultural backgrounds still need to be further deepened. Future research should further explore variables related to “individual and organizational traits,” and view the influence of traditional culture with a more profound and dialectical perspective.

## Data Availability

The raw data supporting the conclusions of this article will be made available by the authors, without undue reservation.

## Footnotes

^1^Data source: Special report on the UN official website. Website: https://news.un.org/zh/story/2022/12/1113042

^2^ZPIN: China’s top professional recruitment service website, with 349 million users in the workplace. Data source: ZPIN Special Report. Website (originally published in Chinese): https://www.fxbaogao.com.

^3^Official standard is a kind of value system that prioritizes officials and regards them as the most important. This value system originated in the Warring States Period (2229 BC–2778 BC), when the thinker Yang Shang proposed it. The official culture was based on the idea that officials should be placed at the top of everything. This value system was prevalent in ancient China and was deeply rooted in the society. It was also the foundation for the establishment of the imperial system.

^4^Harmony is precious, a principle emphasized by Confucianism in the context of moral practice. Specifically, the essence of ritual functions lies in fostering harmony. This implies that when conducting affairs in accordance with rituals, it is essential to appropriately regulate and adjust various interpersonal relationships, thereby enabling individuals to coexist harmoniously.

^5^Percentage change in workplace bullying = $(\frac{\beta *\text{The standard deviation of the independent variable}}{\text{The mean value of the dependent variable}})*100\%=(\frac{0.558*0.852}{1.522})*100\%\approx 31\%.$
